# An Ni-based coordination polymer with a bamboo-like crystal structure

**DOI:** 10.1107/S2056989025002993

**Published:** 2025-04-08

**Authors:** Yi Li, Wang Xie, Chen Lin

**Affiliations:** a Nanjing Petmedicine Technology Co., Ltd, Nanjing, People’s Republic of China; b School of Chemistry and Chemical Engineering Nanjing University,Nanjing 210023 People’s Republic of China; Institute of Chemistry, Chinese Academy of Sciences

**Keywords:** crystal structure, Ni-based coordination polymer, sing-crystal, chain structure

## Abstract

An Ni-based coordination polymer, namely, poly[tetra­aqua­bis­[4,7-bis­(1*H*-pyrazol-4-yl)benzo[*c*][1,2,5]thia­diazole][μ_4_-5,5′-(1,3,6,8-tetra­oxo-1,3,6,8-tetra­hydro­benzo[*lmn*][3,8]phenanthroline-2,7-di­yl)diisophthalato]dinickel(II)], {[Ni_2_(C_30_H_10_N_2_O_12_)(C_12_H_8_N_6_S)_2_(H_2_O)_4_]·2C_3_H_7_NO·H_2_O}_*n*_ or **Ni-BIBT-BINDI**, with a crystal structure reminiscent of bamboo has been synthesized, the Ni^2+^ ions exhibiting a slightly distorted octa­hedral coordination geometry with N atoms and O atoms. The bond lengths of the Ni—O bonds range from 2.032 to 2.121 Å, and those of the Ni—N bonds are approximately 2.080 Å. The BINDI ligands are connected to each other by the Ni—O bonds, which form the backbone of the bamboo, while the BIBT ligands are connected to the backbone of the bamboo through Ni—N bonds and grows on both sides of the bamboo, constituting the bamboo leaves.

## Abstract 2

An Ni-based coordination polymer with a crystal structure reminiscent of bamboo has been synthesized, the Ni^2+^ ions exhibiting a slightly distorted octa­hedral coordination geometry with N atoms and O atoms.

## 1. Chemical context

Coordination polymers are defined as polymers formed by the association of inorganic metal ions and organic ligands with coordination bonds (Xia *et al.*, 2022 ▸). Currently, coordination polymers are being widely used in the fields of catalysis (Li *et al.*, 2024 ▸), sensing (Tian *et al.*, 2023 ▸) and gas storage (Dong *et al.*, 2023 ▸). In particular, Ni-based coordination polymers have received much attention from researchers in recent years because of their excellent catalytic properties (Shah *et al.*, 2019 ▸) and electrical conductivity (Khokhar *et al.*, 2022 ▸).
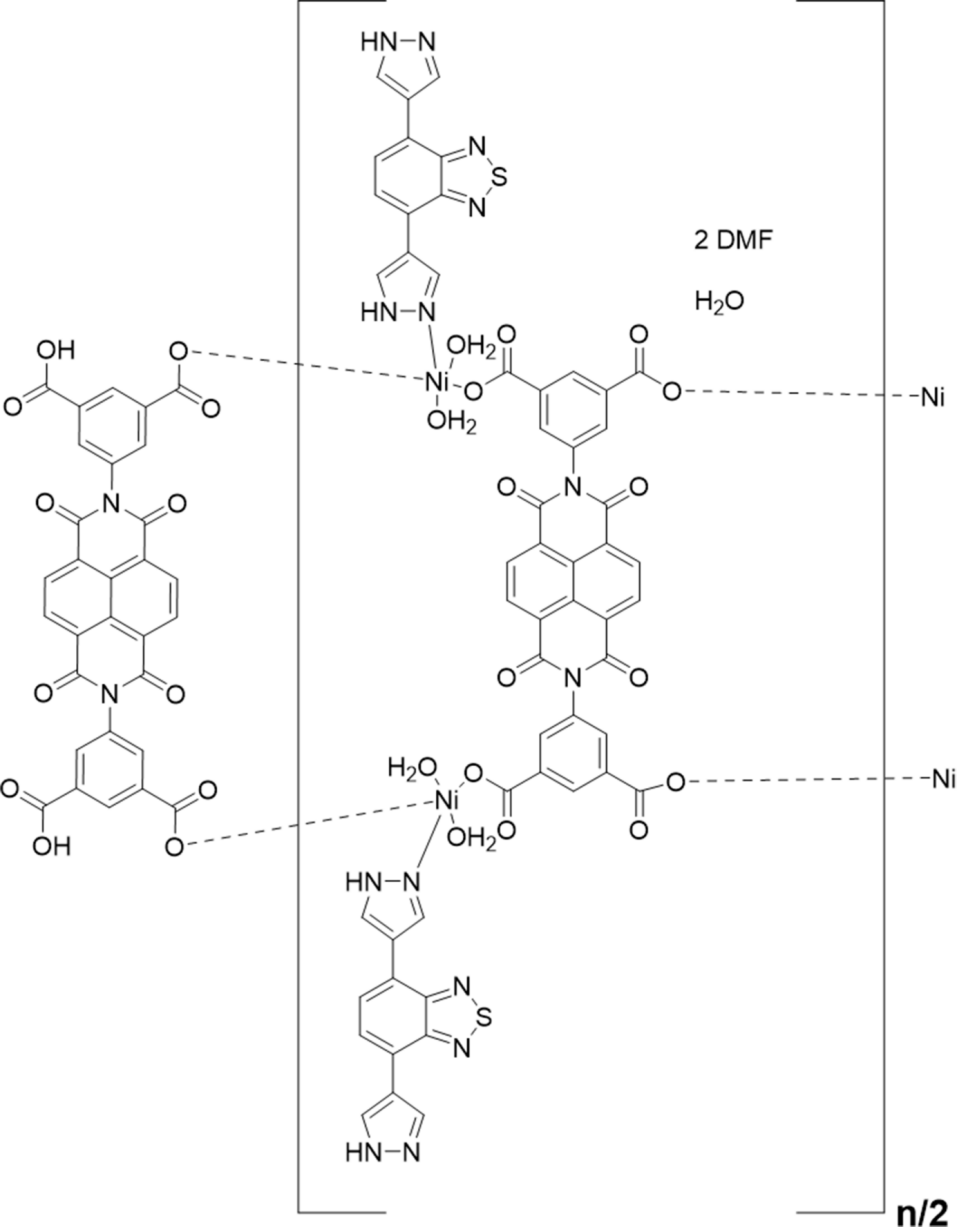


In this context, a novel one-dimensional Ni-based coordination polymer with the formula {[Ni (BINDI)_0.5_(BIBT)(H_2_O)_2_]·DMF·0.5H_2_O]}_*n*_ [DMF = *N,N*-di­methyl­formamide, BIBT = 4,7-di(1*H*-pyrazol-4-yl)benzo[*c*][1,2,5]thia­diazole and H_4_BINDI = 5,5′-(1,3,6,8-tetra­oxo-1,3,6,8-tetra­hydro­benzo[lmn][3,8]phenanthroline-2,7-di­yl)diisophthal­ic acid], namely **Ni-BIBT-BINDI**, was synthesized. The crystal structure of **Ni-BIBT-BINDI** exhibits a bamboo-like morphology, with BINDI^4−^ ions and Ni^2+^ ions connected to form the bamboo trunk, and BIBT ligands and Ni^2+^ ions coordinated to form the bamboo leaves.

## 2. Structural commentary

**Figure 1:**
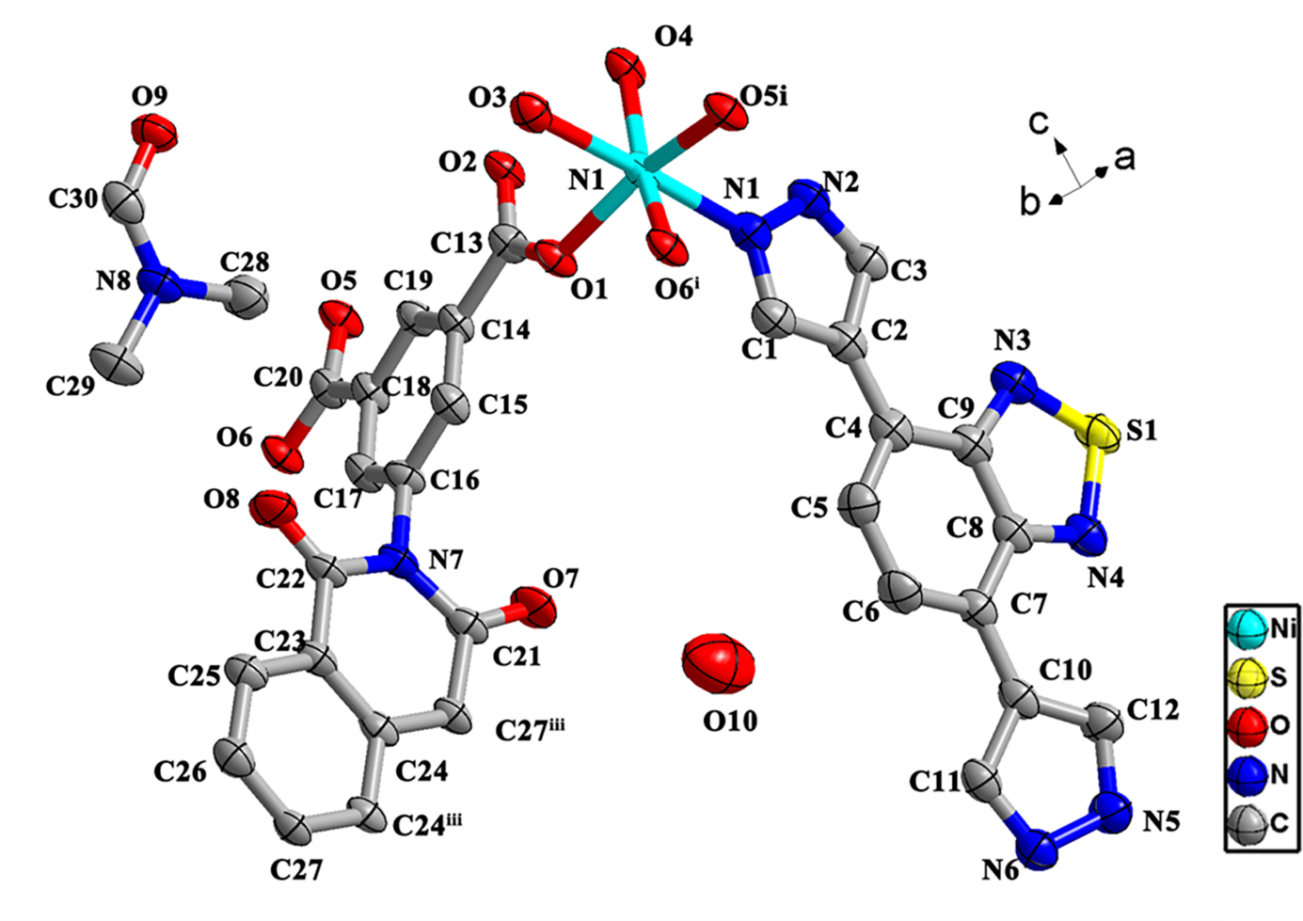
[The coordination environment of **Ni-BIBT-BINDI**, Symmetry codes: (i) *x*, *y* − 1,*z*?(ii) *x*, *y* + 1, *z*; (iii) −*x*, −*y* + 2, −*z*]

**Figure 2:**
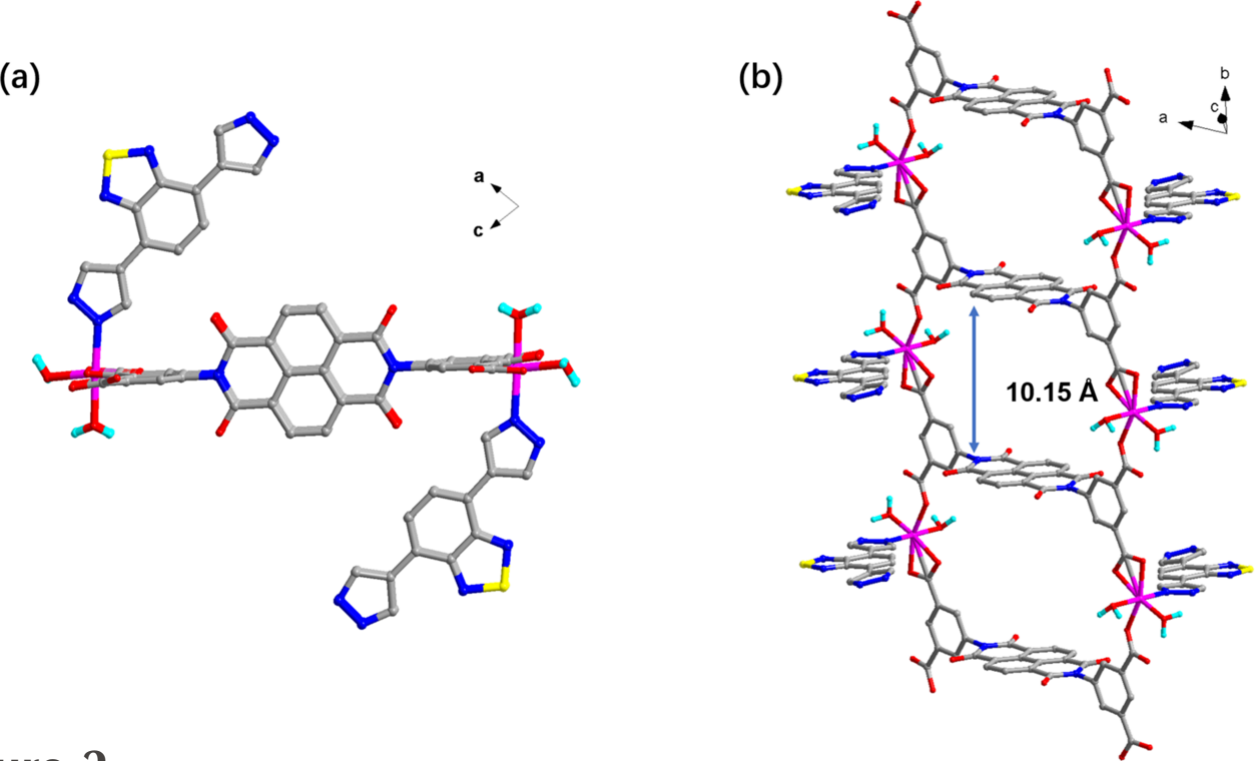
[(*a*)The Ni—BT-BINDI chain was observed from the *b* axis direction. (*b*) The Ni—BT-BINDI chain exhibits a structural similarity to bamboo, with nodes positioned at inter­vals of 10.15 Å]

**Ni-BIBT-BINDI** crystallizes in the triclinic crystal system, space group *P*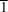. The asymmetric unit contains one Ni^II^ ion, one BIBT ligand and half a BINDI^4−^ ion, one DMF mol­ecule, as well as two coordinated H_2_O mol­ecules and half of a free H_2_O mol­ecule (Fig. 1 ▸). The Ni^II^ ion is surrounded by one nitro­gen atom (N1) from one BIBT mol­ecule, two oxygen atoms (O3, O4) from two different H_2_O mol­ecules, and three oxygen (O1, O5, O6) atoms from two different BINDI^4−^ ions (Fig. 1 ▸), resulting in a distorted octa­hedral coordination geometry (Fig. 1 ▸). The Ni—O bond lengths range from 2.032 (3) to 2.122 (3) Å, while the Ni—N bond length is 2.079 (4) Å (Table 1 ▸), which is consistent with previously reported Ni-based coordination complexes (Wang *et al.*, 2020 ▸). The BINDI^4−^ ions are linked by the Ni^2+^ ions, extending along the *b*-axis direction (Fig. 2 ▸*a*), forming a structure comparable to that of bamboo, while the naphthalene di­imide functional group is almost perpendicular to the *b*-axis, resembling a bamboo joint (Fig. 2 ▸*b*). The distance between adjacent naphthalene di­imide functional groups is 10.15 Å (Fig. 2 ▸*b*). The N atom at the terminal end of the BIBT ligand on the bamboo coordinates with the Ni^2+^ ion and grows in a manner analogous to a bamboo leaf (Fig. 2 ▸*b*). It has been established that only one end of the N atom of the BIBT ligand is coordinated to the Ni^2+^ ion, thus resulting in the formation of a mono-periodic chain.

## 3. Supra­molecular features

**Figure 3:**
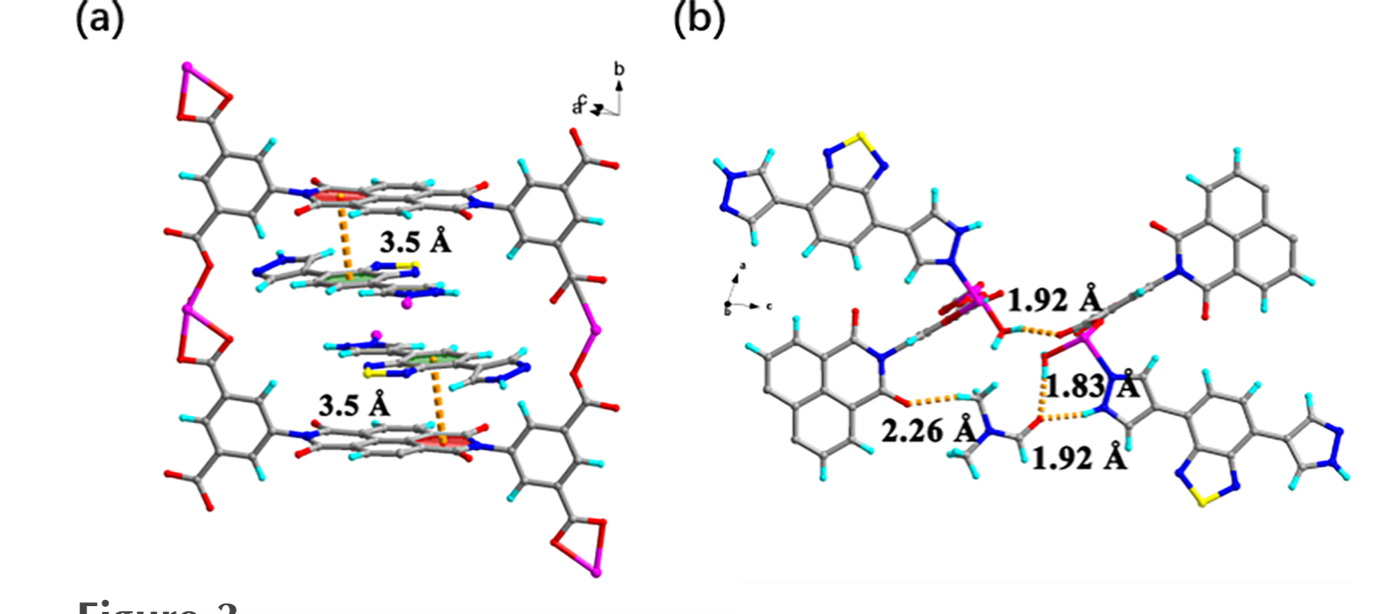
[(*a*) The π-π stacking inter­actions between the ligand BIBT and BINDI, (*b*) The H-bond inter­actions between a DMF mol­ecule and two distinct Ni—BT-BINDI chains.]

In the crystal structure of **Ni-BIBT-BINDI**, the coordination polymer chains are oriented along the *b*-axis direction. The chains are linked by face-to-face π–π stacking inter­actions [centroid–centroid distance = 3.515 (3) Å] between the BIBT ligand and BINDI ion, as shown in Fig. 3 ▸*a*. Within an Ni-BIBT-BINDI chain, the pore between two neighbouring ligand BINDIs contains two ligand BIBTs, which are from different Ni-BIBT-BINDI chains. The distance between the BIBT ligand and the neighbouring BINDI ligand is 3.5 Å, as shown in Fig. 3 ▸*b.* In addition to these π–π stacking inter­actions, there is also evidence of hydrogen-bonding inter­actions between a DMF mol­ecule and two distinct Ni-BIBT-BINDI chains (C28—H28*C*⋯O8, 2.26 Å; O4—H4*B*⋯O9^ii^, 1.83 Å; N2—H2⋯O9^ii^, 1.92 Å; Table 2 ▸). Hydrogen bonding is also present between two different Ni-BIBT-BINDI chains (O3—H3*A*⋯O2^ii^; Fig. 3 ▸*b*, Table 2 ▸). These weak inter­actions connect Ni-BIBT-BINDI chains and build the 3D framework structure of **Ni-BIBT-BINDI**.

## 4. Database survey

A search in CSD (version 5.46, last update November 2024; Groom *et al.*, 2016 ▸) using CONQUEST (Bruno *et al.*, 2002 ▸) for compounds based on BIBT and BINDI ligands revealed that no identical compounds have been reported. However, there are two metal–organic frameworks assembled from ligand BT [4,7-di(1*H*-benzoimidazol-1-yl)benzo[*c*][1,2,5]thia­diazole, structurally analogous to BIBT] in combination with BINDI ligands. The structural unit of the first compound includes Ni^2+^ ions, BINDI and BT ligands (BODCOQ; Xiong *et al.*, 2024 ▸),while that of the second compound includes Cu^2+^ ions, BINDI and BT ligands (TILTHU; Huang *et al.*, 2023 ▸).

## 5. Synthesis and crystallization

The crystal of **Ni-BIBT-BINDI** was synthesized by the solvothermal method. 2.7 mg of BIBT, 6 mg of BINDI, 58 mg of Ni(NO_3_)_2_·6H_2_O, 3.5 mL of DMF and 2.5 mL of deionized water were added in a 10 mL glass tube. After sonication for about 10 min, the glass tube was sealed and heated at 368 K for 24 h. After cooling to room temperature, green crystals were collected.

## 6. Refinement

Crystal data, data collection and structure refinement details are summarized in Table 3 ▸. H atoms were positioned geometrically and refined using a riding model.

## Supplementary Material

Crystal structure: contains datablock(s) I. DOI: 10.1107/S2056989025002993/nx2022sup1.cif

Structure factors: contains datablock(s) I. DOI: 10.1107/S2056989025002993/nx2022Isup3.hkl

CCDC reference: 2364669

Additional supporting information:  crystallographic information; 3D view; checkCIF report

Additional supporting information:  crystallographic information; 3D view; checkCIF report

## Figures and Tables

**Table 1:** Selected geometric parameters (Å, °)

Ni1—O1	2.042 (3)	Ni1—O5^i^	2.115 (3)
Ni1—O3	2.101 (3)	Ni1—O6^i^	2.122 (3)
Ni1—O4	2.032 (3)	Ni1—N1	2.079 (4)
			
O1—Ni1—O3	89.17 (14)	O4—Ni1—O5^i^	103.05 (13)
O1—Ni1—O5^i^	160.93 (13)	O4—Ni1—O6^i^	164.09 (13)
O1—Ni1—O6^i^	98.70 (12)	O4—Ni1—N1	96.72 (15)
O1—Ni1—N1	89.40 (15)	O5^i^—Ni1—O6^i^	62.43 (12)
O3—Ni1—O5^i^	87.54 (13)	N1—Ni1—O3	178.02 (14)
O3—Ni1—O6^i^	87.96 (13)	N1—Ni1—O5^i^	93.37 (15)
O4—Ni1—O1	95.34 (13)	N1—Ni1—O6^i^	90.90 (15)
O4—Ni1—O3	84.78 (14)		

Symmetry code: (i) 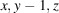.

**Table 2:** Hydrogen-bond geometry (Å, °)

*D*—H⋯*A*	*D*—H	H⋯*A*	*D*⋯*A*	*D*—H⋯*A*
O3—H3*A*⋯O2^ii^	0.87	1.92	2.758 (5)	162
O3—H3*B*⋯N6^iii^	0.87	2.18	2.986 (5)	154
O4—H4*A*⋯O2	0.87	1.86	2.640 (4)	148
O4—H4*B*⋯O9^ii^	0.87	1.83	2.695 (5)	173
N2—H2⋯O9^ii^	0.88	1.92	2.779 (5)	166
N5—H5⋯N6^iv^	0.88	2.20	2.956 (6)	144
C1—H1⋯O10^iii^	0.95	2.57	3.46 (3)	155
C11—H11⋯O1^iii^	0.95	2.54	3.210 (6)	127
C11—H11⋯O6^v^	0.95	2.49	3.279 (6)	141
C12—H12⋯O2^vi^	0.95	2.46	3.168 (6)	131
C28—H28*A*⋯N5^iii^	0.98	2.65	3.426 (8)	136
C28—H28*B*⋯O4^ii^	0.98	2.72	3.414 (6)	128
C28—H28*C*⋯O8	0.98	2.26	3.157 (7)	151
O10—H10*A*⋯O7	0.87	2.08	2.95 (3)	180
O10—H10*B*⋯O6^v^	0.87	2.20	3.01 (3)	155

Symmetry codes: (ii) 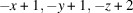; (iii) 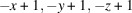; (iv) 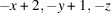; (v) 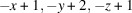; (vi) 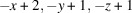.

**Table 3:** Experimental details

Crystal data	
Chemical formula	[Ni_2_(C_30_H_10_N_2_O_12_)(C_12_H_8_N_6_S)_2_(H_2_O)_4_]·2(C_3_H_7_NO)·H_2_O
*M*_r_	1480.70
Crystal system, space group	Triclinic, *P* 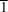
Temperature (K)	193
*a*, *b*, *c* (Å)	9.5384 (4), 10.1543 (4), 16.2521 (8)
α, β, γ (°)	81.511 (3), 75.790 (3), 75.920 (3)
*V* (Å^3^)	1473.62 (12)
*Z*	1
Radiation type	Cu *K*α
μ (mm^−1^)	2.27
Crystal size (mm)	0.16 × 0.12 × 0.11

Data collection	
Diffractometer	Bruker PHOTON-II area detector
Absorption correction	Multi-scan (*SADABS*; Krause *et al.*, 2015 ▸)
*T*_min_, *T*_max_	0.544, 0.753
No. of measured, independent and observed [*I* > 2σ(*I*)] reflections	24224, 5414, 4005
*R*_int_	0.068
(sin θ/λ)_max_ (Å^−1^)	0.604

Refinement	
*R*[*F*^2^ > 2σ(*F*^2^)], *wR*(*F*^2^), *S*	0.076, 0.247, 1.06
No. of reflections	5414
No. of parameters	455
H-atom treatment	H-atom parameters constrained
Δρ_max_, Δρ_min_ (e Å^−3^)	1.46, −1.04

Computer programs: *APEX2* and *SAINT* (Bruker, 2016 ▸), *OLEX2.solve* (Bourhis *et al.*, 2015 ▸), *SHELXL2018/3* (Sheldrick, 2015 ▸) and *OLEX2* (Dolomanov *et al.*, 2009 ▸).

## supplementary crystallographic information

### Poly[tetraaquabis[4,7-bis(1H-pyrazol-4-yl)benzo[c][1,2,5]thiadiazole][µ4-5,5'-(1,3,6,8-tetraoxo-1,3,6,8-tetrahydrobenzo[lmn][3,8]phenanthroline-2,7-diyl)diisophthalato]dinickel(II)] Crystal data

**Table:**

[Ni_2_(C_30_H_10_N_2_O_12_)(C_12_H_8_N_6_S)_2_(H_2_O)_4_]·2(C_3_H_7_NO)·H_2_O	*Z* = 1
*M**_r_* = 1480.70	*F*(000) = 762
Triclinic, *P*1	*D*_x_ = 1.669 Mg m^−^^3^
*a* = 9.5384 (4) Å	Cu *K*α radiation, λ = 1.54178 Å
*b* = 10.1543 (4) Å	Cell parameters from 7699 reflections
*c* = 16.2521 (8) Å	θ = 4.5–68.1°
α = 81.511 (3)°	µ = 2.27 mm^−^^1^
β = 75.790 (3)°	*T* = 193 K
γ = 75.920 (3)°	Block, dull greenish green
*V* = 1473.62 (12) Å^3^	0.16 × 0.12 × 0.11 mm

### Poly[tetraaquabis[4,7-bis(1H-pyrazol-4-yl)benzo[c][1,2,5]thiadiazole][µ4-5,5'-(1,3,6,8-tetraoxo-1,3,6,8-tetrahydrobenzo[lmn][3,8]phenanthroline-2,7-diyl)diisophthalato]dinickel(II)] Data collection

**Table:**

Bruker PHOTON-II area detector diffractometer	4005 reflections with *I* > 2σ(*I*)
Detector resolution: 7.4 pixels mm^-1^	*R*_int_ = 0.068
phi and ω scans	θ_max_ = 68.6°, θ_min_ = 2.8°
Absorption correction: multi-scan (SADABS; Krause *et al.*, 2015)	*h* = −11→11
*T*_min_ = 0.544, *T*_max_ = 0.753	*k* = −12→12
24224 measured reflections	*l* = −19→19
5414 independent reflections	

### Poly[tetraaquabis[4,7-bis(1H-pyrazol-4-yl)benzo[c][1,2,5]thiadiazole][µ4-5,5'-(1,3,6,8-tetraoxo-1,3,6,8-tetrahydrobenzo[lmn][3,8]phenanthroline-2,7-diyl)diisophthalato]dinickel(II)] Refinement

**Table:**

Refinement on *F*^2^	0 restraints
Least-squares matrix: full	Hydrogen site location: mixed
*R*[*F*^2^ > 2σ(*F*^2^)] = 0.076	H-atom parameters constrained
*wR*(*F*^2^) = 0.247	*w* = 1/[σ^2^(*F*_o_^2^) + (0.1781*P*)^2^ + 0.7895*P*] where *P* = (*F*_o_^2^ + 2*F*_c_^2^)/3
*S* = 1.06	(Δ/σ)_max_ < 0.001
5414 reflections	Δρ_max_ = 1.46 e Å^−^^3^
455 parameters	Δρ_min_ = −1.04 e Å^−^^3^

### Poly[tetraaquabis[4,7-bis(1H-pyrazol-4-yl)benzo[c][1,2,5]thiadiazole][µ4-5,5'-(1,3,6,8-tetraoxo-1,3,6,8-tetrahydrobenzo[lmn][3,8]phenanthroline-2,7-diyl)diisophthalato]dinickel(II)] Special details

**Table:**

Geometry. All esds (except the esd in the dihedral angle between two l.s. planes) are estimated using the full covariance matrix. The cell esds are taken into account individually in the estimation of esds in distances, angles and torsion angles; correlations between esds in cell parameters are only used when they are defined by crystal symmetry. An approximate (isotropic) treatment of cell esds is used for estimating esds involving l.s. planes.

### Poly[tetraaquabis[4,7-bis(1H-pyrazol-4-yl)benzo[c][1,2,5]thiadiazole][µ4-5,5'-(1,3,6,8-tetraoxo-1,3,6,8-tetrahydrobenzo[lmn][3,8]phenanthroline-2,7-diyl)diisophthalato]dinickel(II)] Fractional atomic coordinates and isotropic or equivalent isotropic displacement parameters (Å^2^)

**Table:**

	*x*	*y*	*z*	*U*_iso_*/*U*_eq_	Occ. (<1)
Ni1	0.57153 (8)	0.31000 (7)	0.84217 (5)	0.0304 (3)	
S1	1.29018 (14)	0.30007 (14)	0.39030 (9)	0.0450 (4)	
O1	0.4971 (4)	0.5137 (3)	0.8127 (2)	0.0341 (7)	
O2	0.5865 (3)	0.6001 (3)	0.9027 (2)	0.0335 (7)	
O3	0.3923 (4)	0.3157 (3)	0.9478 (2)	0.0378 (8)	
H3A	0.418410	0.332432	0.992361	0.057*	
H3B	0.321320	0.385253	0.939452	0.057*	
O4	0.6859 (4)	0.3342 (3)	0.9272 (2)	0.0359 (7)	
H4A	0.680119	0.420791	0.928462	0.054*	
H4B	0.779938	0.300760	0.908602	0.054*	
O5	0.5926 (4)	1.0963 (3)	0.8525 (2)	0.0349 (7)	
O6	0.4480 (4)	1.2335 (3)	0.7751 (2)	0.0337 (7)	
O7	0.4323 (4)	0.8766 (4)	0.5411 (2)	0.0492 (9)	
O8	0.0255 (4)	0.9415 (4)	0.7567 (2)	0.0496 (10)	
N1	0.7439 (4)	0.3085 (4)	0.7351 (3)	0.0359 (9)	
N2	0.8912 (5)	0.2834 (4)	0.7296 (3)	0.0393 (9)	
H2	0.933374	0.260189	0.773567	0.047*	
N3	1.1602 (5)	0.3014 (4)	0.4758 (3)	0.0423 (10)	
N4	1.1933 (5)	0.3483 (4)	0.3182 (3)	0.0408 (10)	
N5	1.0322 (5)	0.4553 (5)	0.0894 (3)	0.0454 (10)	
H5	1.094565	0.446406	0.039685	0.055*	
N6	0.8855 (5)	0.5163 (5)	0.0989 (3)	0.0416 (10)	
N7	0.2282 (4)	0.9214 (4)	0.6484 (2)	0.0311 (8)	
C1	0.7268 (6)	0.3386 (5)	0.6562 (3)	0.0396 (11)	
H1	0.633402	0.359982	0.640809	0.048*	
C2	0.8620 (5)	0.3357 (5)	0.5972 (3)	0.0358 (10)	
C3	0.9646 (6)	0.2981 (5)	0.6490 (3)	0.0372 (10)	
H3	1.069080	0.285286	0.630045	0.045*	
C4	0.8869 (5)	0.3612 (5)	0.5052 (3)	0.0363 (10)	
C5	0.7710 (6)	0.4059 (5)	0.4641 (3)	0.0410 (11)	
H5A	0.672881	0.419811	0.497727	0.049*	
C6	0.7905 (6)	0.4322 (6)	0.3745 (3)	0.0434 (12)	
H6	0.704894	0.463219	0.351132	0.052*	
C7	0.9277 (5)	0.4149 (5)	0.3194 (3)	0.0346 (10)	
C8	1.0512 (6)	0.3685 (5)	0.3594 (3)	0.0356 (10)	
C9	1.0309 (6)	0.3426 (5)	0.4498 (3)	0.0361 (10)	
C10	0.9456 (5)	0.4426 (5)	0.2269 (3)	0.0368 (10)	
C11	0.8347 (6)	0.5097 (5)	0.1819 (3)	0.0393 (11)	
H11	0.734986	0.545945	0.208585	0.047*	
C12	1.0702 (6)	0.4106 (6)	0.1641 (3)	0.0434 (12)	
H12	1.166018	0.364911	0.172088	0.052*	
C13	0.5182 (5)	0.6112 (4)	0.8442 (3)	0.0292 (9)	
C14	0.4599 (5)	0.7526 (4)	0.8049 (3)	0.0293 (9)	
C15	0.3696 (5)	0.7719 (4)	0.7469 (3)	0.0313 (9)	
H15	0.340778	0.696185	0.732957	0.038*	
C16	0.3218 (5)	0.9009 (5)	0.7094 (3)	0.0320 (10)	
C17	0.3612 (5)	1.0118 (4)	0.7286 (3)	0.0323 (10)	
H17	0.326368	1.100122	0.702890	0.039*	
C18	0.4529 (5)	0.9944 (4)	0.7863 (3)	0.0295 (9)	
C19	0.5010 (5)	0.8645 (4)	0.8249 (3)	0.0306 (9)	
H19	0.561972	0.852347	0.864919	0.037*	
C20	0.5006 (5)	1.1154 (4)	0.8060 (3)	0.0309 (9)	
C21	0.2983 (5)	0.9141 (5)	0.5622 (3)	0.0344 (10)	
C22	0.0752 (5)	0.9435 (5)	0.6811 (3)	0.0323 (10)	
C23	−0.0198 (5)	0.9732 (4)	0.6173 (3)	0.0314 (10)	
C24	0.0452 (5)	0.9811 (4)	0.5302 (3)	0.0298 (9)	
C25	−0.1718 (5)	0.9988 (5)	0.6451 (3)	0.0358 (10)	
H25	−0.214907	0.991401	0.704439	0.043*	
C26	−0.2624 (6)	1.0358 (5)	0.5860 (3)	0.0381 (11)	
H26	−0.367033	1.053781	0.605420	0.046*	
C27	−0.2012 (5)	1.0462 (4)	0.5001 (3)	0.0309 (9)	
O9	0.0216 (4)	0.7746 (4)	1.1156 (2)	0.0448 (9)	
N8	−0.0606 (5)	0.8531 (5)	0.9943 (3)	0.0427 (10)	
C28	0.0864 (7)	0.8243 (7)	0.9386 (4)	0.0523 (14)	
H28A	0.112379	0.728549	0.926460	0.078*	
H28B	0.159091	0.841639	0.966780	0.078*	
H28C	0.086624	0.883241	0.885104	0.078*	
C29	−0.1853 (7)	0.9179 (6)	0.9545 (4)	0.0526 (14)	
H29A	−0.168246	1.004746	0.923080	0.079*	
H29B	−0.276681	0.934675	0.998658	0.079*	
H29C	−0.194565	0.857833	0.915068	0.079*	
C30	−0.0785 (6)	0.8241 (5)	1.0773 (3)	0.0415 (11)	
H30	−0.176878	0.842806	1.110522	0.050*	
O10	0.584 (3)	0.692 (2)	0.4066 (17)	0.217 (12)	0.5
H10A	0.539592	0.746254	0.446417	0.326*	0.5
H10B	0.557502	0.736204	0.360787	0.326*	0.5

### Poly[tetraaquabis[4,7-bis(1H-pyrazol-4-yl)benzo[c][1,2,5]thiadiazole][µ4-5,5'-(1,3,6,8-tetraoxo-1,3,6,8-tetrahydrobenzo[lmn][3,8]phenanthroline-2,7-diyl)diisophthalato]dinickel(II)] Atomic displacement parameters (Å^2^)

**Table:**

	*U*^11^	*U*^22^	*U*^33^	*U*^12^	*U*^13^	*U*^23^
Ni1	0.0285 (5)	0.0232 (4)	0.0429 (5)	−0.0058 (3)	−0.0149 (3)	−0.0012 (3)
S1	0.0326 (7)	0.0497 (8)	0.0521 (8)	−0.0048 (5)	−0.0156 (5)	0.0007 (6)
O1	0.0359 (18)	0.0220 (15)	0.0500 (19)	−0.0064 (13)	−0.0215 (14)	−0.0001 (13)
O2	0.0311 (17)	0.0249 (15)	0.0477 (18)	−0.0025 (13)	−0.0198 (14)	−0.0004 (13)
O3	0.0331 (17)	0.0382 (18)	0.0457 (19)	−0.0060 (14)	−0.0160 (14)	−0.0057 (14)
O4	0.0321 (17)	0.0288 (16)	0.0497 (19)	−0.0045 (13)	−0.0172 (14)	−0.0027 (13)
O5	0.0336 (17)	0.0254 (16)	0.0515 (19)	−0.0069 (13)	−0.0217 (14)	−0.0001 (13)
O6	0.0335 (17)	0.0216 (15)	0.0504 (19)	−0.0044 (12)	−0.0202 (14)	−0.0015 (13)
O7	0.0244 (18)	0.069 (3)	0.054 (2)	−0.0009 (17)	−0.0190 (15)	−0.0023 (18)
O8	0.0354 (19)	0.071 (3)	0.042 (2)	−0.0040 (18)	−0.0182 (15)	0.0006 (17)
N1	0.032 (2)	0.033 (2)	0.045 (2)	−0.0080 (16)	−0.0119 (17)	−0.0034 (16)
N2	0.037 (2)	0.036 (2)	0.047 (2)	−0.0058 (18)	−0.0170 (18)	−0.0021 (17)
N3	0.042 (2)	0.036 (2)	0.052 (2)	−0.0081 (18)	−0.0181 (19)	0.0003 (18)
N4	0.032 (2)	0.038 (2)	0.054 (3)	−0.0078 (17)	−0.0136 (18)	−0.0016 (18)
N5	0.036 (2)	0.057 (3)	0.042 (2)	−0.010 (2)	−0.0089 (18)	−0.0025 (19)
N6	0.035 (2)	0.046 (2)	0.045 (2)	−0.0084 (19)	−0.0124 (18)	−0.0032 (18)
N7	0.0269 (19)	0.0271 (18)	0.043 (2)	−0.0060 (15)	−0.0174 (15)	0.0019 (15)
C1	0.040 (3)	0.034 (2)	0.048 (3)	−0.007 (2)	−0.017 (2)	−0.003 (2)
C2	0.035 (3)	0.028 (2)	0.047 (3)	−0.0097 (19)	−0.014 (2)	−0.0032 (19)
C3	0.035 (3)	0.034 (2)	0.045 (3)	−0.009 (2)	−0.014 (2)	−0.0008 (19)
C4	0.035 (3)	0.030 (2)	0.049 (3)	−0.0120 (19)	−0.013 (2)	−0.0020 (19)
C5	0.031 (3)	0.047 (3)	0.047 (3)	−0.011 (2)	−0.008 (2)	−0.004 (2)
C6	0.033 (3)	0.049 (3)	0.053 (3)	−0.008 (2)	−0.018 (2)	−0.006 (2)
C7	0.030 (2)	0.031 (2)	0.045 (3)	−0.0070 (19)	−0.0131 (19)	−0.0036 (19)
C8	0.034 (2)	0.028 (2)	0.046 (3)	−0.0090 (19)	−0.012 (2)	−0.0013 (19)
C9	0.037 (3)	0.028 (2)	0.048 (3)	−0.0105 (19)	−0.015 (2)	−0.0026 (19)
C10	0.030 (2)	0.033 (2)	0.049 (3)	−0.0072 (19)	−0.014 (2)	−0.002 (2)
C11	0.032 (2)	0.037 (3)	0.051 (3)	−0.005 (2)	−0.014 (2)	−0.005 (2)
C12	0.033 (3)	0.052 (3)	0.047 (3)	−0.008 (2)	−0.014 (2)	−0.003 (2)
C13	0.026 (2)	0.024 (2)	0.041 (2)	−0.0089 (17)	−0.0120 (17)	0.0002 (17)
C14	0.023 (2)	0.024 (2)	0.041 (2)	−0.0011 (17)	−0.0110 (17)	−0.0022 (17)
C15	0.026 (2)	0.025 (2)	0.046 (3)	−0.0068 (17)	−0.0114 (18)	−0.0031 (18)
C16	0.029 (2)	0.031 (2)	0.040 (2)	−0.0063 (18)	−0.0171 (18)	−0.0003 (18)
C17	0.028 (2)	0.023 (2)	0.044 (3)	−0.0019 (17)	−0.0125 (18)	0.0003 (17)
C18	0.022 (2)	0.023 (2)	0.046 (2)	−0.0056 (16)	−0.0113 (17)	−0.0028 (17)
C19	0.026 (2)	0.029 (2)	0.039 (2)	−0.0058 (17)	−0.0133 (18)	−0.0024 (18)
C20	0.026 (2)	0.026 (2)	0.041 (2)	−0.0044 (17)	−0.0091 (18)	−0.0024 (17)
C21	0.032 (2)	0.030 (2)	0.046 (3)	−0.0068 (19)	−0.0196 (19)	−0.0001 (18)
C22	0.029 (2)	0.029 (2)	0.043 (3)	−0.0054 (18)	−0.0180 (19)	0.0018 (18)
C23	0.028 (2)	0.025 (2)	0.044 (2)	−0.0060 (18)	−0.0168 (19)	−0.0001 (17)
C24	0.028 (2)	0.021 (2)	0.044 (2)	−0.0057 (17)	−0.0159 (19)	0.0002 (17)
C25	0.031 (2)	0.034 (2)	0.043 (3)	−0.0076 (19)	−0.0123 (19)	0.0021 (19)
C26	0.029 (2)	0.038 (3)	0.047 (3)	−0.005 (2)	−0.012 (2)	−0.001 (2)
C27	0.025 (2)	0.028 (2)	0.043 (2)	−0.0047 (17)	−0.0150 (18)	−0.0008 (17)
O9	0.0387 (19)	0.050 (2)	0.047 (2)	−0.0044 (16)	−0.0193 (16)	0.0019 (15)
N8	0.038 (2)	0.044 (2)	0.048 (2)	−0.0057 (19)	−0.0194 (19)	0.0007 (18)
C28	0.043 (3)	0.062 (4)	0.049 (3)	−0.007 (3)	−0.014 (2)	0.001 (3)
C29	0.046 (3)	0.050 (3)	0.065 (4)	−0.005 (3)	−0.029 (3)	0.006 (3)
C30	0.032 (3)	0.038 (3)	0.056 (3)	−0.008 (2)	−0.013 (2)	−0.001 (2)
O10	0.29 (3)	0.16 (2)	0.22 (2)	−0.02 (2)	−0.11 (2)	−0.028 (17)

### Poly[tetraaquabis[4,7-bis(1H-pyrazol-4-yl)benzo[c][1,2,5]thiadiazole][µ4-5,5'-(1,3,6,8-tetraoxo-1,3,6,8-tetrahydrobenzo[lmn][3,8]phenanthroline-2,7-diyl)diisophthalato]dinickel(II)] Geometric parameters (Å, º)

**Table:**

Ni1—O1	2.042 (3)	C6—C7	1.380 (7)
Ni1—O3	2.101 (3)	C7—C8	1.432 (7)
Ni1—O4	2.032 (3)	C7—C10	1.462 (7)
Ni1—O5^i^	2.115 (3)	C8—C9	1.428 (7)
Ni1—O6^i^	2.122 (3)	C10—C11	1.415 (7)
Ni1—N1	2.079 (4)	C10—C12	1.370 (7)
Ni1—C20^i^	2.428 (5)	C11—H11	0.9500
S1—N3	1.618 (5)	C12—H12	0.9500
S1—N4	1.615 (4)	C13—C14	1.515 (6)
O1—C13	1.257 (5)	C14—C15	1.390 (6)
O2—C13	1.257 (5)	C14—C19	1.393 (6)
O3—H3A	0.8717	C15—H15	0.9500
O3—H3B	0.8720	C15—C16	1.380 (6)
O4—H4A	0.8703	C16—C17	1.368 (7)
O4—H4B	0.8715	C17—H17	0.9500
O5—C20	1.256 (6)	C17—C18	1.398 (6)
O6—C20	1.263 (5)	C18—C19	1.395 (6)
O7—C21	1.217 (6)	C18—C20	1.510 (6)
O8—C22	1.202 (6)	C19—H19	0.9500
N1—N2	1.348 (6)	C21—C27^ii^	1.484 (6)
N1—C1	1.314 (6)	C22—C23	1.491 (6)
N2—H2	0.8800	C23—C24	1.398 (7)
N2—C3	1.330 (7)	C23—C25	1.380 (7)
N3—C9	1.351 (6)	C24—C24^ii^	1.413 (8)
N4—C8	1.336 (6)	C24—C27^ii^	1.419 (6)
N5—H5	0.8800	C25—H25	0.9500
N5—N6	1.366 (6)	C25—C26	1.397 (7)
N5—C12	1.335 (7)	C26—H26	0.9500
N6—C11	1.314 (7)	C26—C27	1.375 (7)
N7—C16	1.452 (5)	O9—C30	1.232 (6)
N7—C21	1.401 (6)	N8—C28	1.459 (7)
N7—C22	1.399 (6)	N8—C29	1.462 (6)
C1—H1	0.9500	N8—C30	1.315 (7)
C1—C2	1.401 (7)	C28—H28A	0.9800
C2—C3	1.393 (7)	C28—H28B	0.9800
C2—C4	1.450 (7)	C28—H28C	0.9800
C3—H3	0.9500	C29—H29A	0.9800
C4—C5	1.380 (7)	C29—H29B	0.9800
C4—C9	1.431 (7)	C29—H29C	0.9800
C5—H5A	0.9500	C30—H30	0.9500
C5—C6	1.415 (7)	O10—H10A	0.8701
C6—H6	0.9500	O10—H10B	0.8699
			
O1—Ni1—O3	89.17 (14)	C12—C10—C11	103.9 (4)
O1—Ni1—O5^i^	160.93 (13)	N6—C11—C10	112.2 (5)
O1—Ni1—O6^i^	98.70 (12)	N6—C11—H11	123.9
O1—Ni1—N1	89.40 (15)	C10—C11—H11	123.9
O1—Ni1—C20^i^	129.91 (14)	N5—C12—C10	107.5 (5)
O3—Ni1—O5^i^	87.54 (13)	N5—C12—H12	126.3
O3—Ni1—O6^i^	87.96 (13)	C10—C12—H12	126.3
O3—Ni1—C20^i^	86.63 (14)	O1—C13—O2	125.5 (4)
O4—Ni1—O1	95.34 (13)	O1—C13—C14	116.0 (4)
O4—Ni1—O3	84.78 (14)	O2—C13—C14	118.5 (4)
O4—Ni1—O5^i^	103.05 (13)	C15—C14—C13	120.9 (4)
O4—Ni1—O6^i^	164.09 (13)	C15—C14—C19	119.4 (4)
O4—Ni1—N1	96.72 (15)	C19—C14—C13	119.7 (4)
O4—Ni1—C20^i^	133.75 (14)	C14—C15—H15	120.0
O5^i^—Ni1—O6^i^	62.43 (12)	C16—C15—C14	120.1 (4)
O5^i^—Ni1—C20^i^	31.13 (13)	C16—C15—H15	120.0
O6^i^—Ni1—C20^i^	31.31 (14)	C15—C16—N7	120.3 (4)
N1—Ni1—O3	178.02 (14)	C17—C16—N7	118.6 (4)
N1—Ni1—O5^i^	93.37 (15)	C17—C16—C15	121.1 (4)
N1—Ni1—O6^i^	90.90 (15)	C16—C17—H17	120.2
N1—Ni1—C20^i^	93.24 (16)	C16—C17—C18	119.7 (4)
N4—S1—N3	100.7 (2)	C18—C17—H17	120.2
C13—O1—Ni1	127.5 (3)	C17—C18—C20	120.1 (4)
Ni1—O3—H3A	109.5	C19—C18—C17	119.6 (4)
Ni1—O3—H3B	109.5	C19—C18—C20	120.3 (4)
H3A—O3—H3B	104.4	C14—C19—C18	120.1 (4)
Ni1—O4—H4A	109.2	C14—C19—H19	119.9
Ni1—O4—H4B	109.3	C18—C19—H19	119.9
H4A—O4—H4B	104.5	O5—C20—Ni1^iii^	60.6 (2)
C20—O5—Ni1^iii^	88.3 (3)	O5—C20—O6	121.4 (4)
C20—O6—Ni1^iii^	87.8 (3)	O5—C20—C18	119.1 (4)
N2—N1—Ni1	129.7 (3)	O6—C20—Ni1^iii^	60.9 (2)
C1—N1—Ni1	124.8 (4)	O6—C20—C18	119.5 (4)
C1—N1—N2	105.4 (4)	C18—C20—Ni1^iii^	178.1 (3)
N1—N2—H2	124.3	O7—C21—N7	120.4 (4)
C3—N2—N1	111.3 (4)	O7—C21—C27^ii^	123.0 (5)
C3—N2—H2	124.3	N7—C21—C27^ii^	116.6 (4)
C9—N3—S1	106.3 (4)	O8—C22—N7	120.7 (4)
C8—N4—S1	106.5 (4)	O8—C22—C23	123.0 (4)
N6—N5—H5	123.8	N7—C22—C23	116.3 (4)
C12—N5—H5	123.8	C24—C23—C22	119.9 (4)
C12—N5—N6	112.4 (4)	C25—C23—C22	119.3 (4)
C11—N6—N5	104.0 (4)	C25—C23—C24	120.7 (4)
C21—N7—C16	117.5 (4)	C23—C24—C24^ii^	119.9 (5)
C22—N7—C16	117.0 (4)	C23—C24—C27^ii^	121.8 (4)
C22—N7—C21	125.5 (4)	C24^ii^—C24—C27^ii^	118.3 (5)
N1—C1—H1	123.8	C23—C25—H25	120.0
N1—C1—C2	112.4 (5)	C23—C25—C26	120.0 (5)
C2—C1—H1	123.8	C26—C25—H25	120.0
C1—C2—C4	128.0 (4)	C25—C26—H26	119.8
C3—C2—C1	102.7 (4)	C27—C26—C25	120.5 (5)
C3—C2—C4	129.3 (5)	C27—C26—H26	119.8
N2—C3—C2	108.2 (5)	C24^ii^—C27—C21^ii^	119.3 (4)
N2—C3—H3	125.9	C26—C27—C21^ii^	120.1 (4)
C2—C3—H3	125.9	C26—C27—C24^ii^	120.6 (4)
C5—C4—C2	121.7 (5)	C28—N8—C29	117.5 (5)
C5—C4—C9	114.7 (5)	C30—N8—C28	120.7 (4)
C9—C4—C2	123.6 (4)	C30—N8—C29	121.8 (5)
C4—C5—H5A	118.2	N8—C28—H28A	109.5
C4—C5—C6	123.5 (5)	N8—C28—H28B	109.5
C6—C5—H5A	118.2	N8—C28—H28C	109.5
C5—C6—H6	118.3	H28A—C28—H28B	109.5
C7—C6—C5	123.3 (5)	H28A—C28—H28C	109.5
C7—C6—H6	118.3	H28B—C28—H28C	109.5
C6—C7—C8	115.0 (4)	N8—C29—H29A	109.5
C6—C7—C10	122.5 (4)	N8—C29—H29B	109.5
C8—C7—C10	122.4 (4)	N8—C29—H29C	109.5
N4—C8—C7	124.9 (5)	H29A—C29—H29B	109.5
N4—C8—C9	113.7 (4)	H29A—C29—H29C	109.5
C9—C8—C7	121.4 (4)	H29B—C29—H29C	109.5
N3—C9—C4	125.1 (5)	O9—C30—N8	125.4 (5)
N3—C9—C8	112.8 (4)	O9—C30—H30	117.3
C8—C9—C4	122.1 (4)	N8—C30—H30	117.3
C11—C10—C7	127.0 (5)	H10A—O10—H10B	104.5
C12—C10—C7	129.1 (4)		
			
Ni1—O1—C13—O2	−2.5 (7)	C7—C8—C9—N3	−179.1 (4)
Ni1—O1—C13—C14	175.2 (3)	C7—C8—C9—C4	0.1 (7)
Ni1^iii^—O5—C20—O6	2.5 (4)	C7—C10—C11—N6	−178.7 (5)
Ni1^iii^—O5—C20—C18	−177.8 (4)	C7—C10—C12—N5	179.4 (5)
Ni1^iii^—O6—C20—O5	−2.5 (4)	C8—C7—C10—C11	−168.5 (5)
Ni1^iii^—O6—C20—C18	177.8 (4)	C8—C7—C10—C12	11.8 (8)
Ni1—N1—N2—C3	−177.3 (3)	C9—C4—C5—C6	−0.1 (7)
Ni1—N1—C1—C2	177.0 (3)	C10—C7—C8—N4	1.4 (8)
S1—N3—C9—C4	−178.5 (4)	C10—C7—C8—C9	179.8 (4)
S1—N3—C9—C8	0.7 (5)	C11—C10—C12—N5	−0.4 (6)
S1—N4—C8—C7	178.6 (4)	C12—N5—N6—C11	1.0 (6)
S1—N4—C8—C9	0.1 (5)	C12—C10—C11—N6	1.1 (6)
O1—C13—C14—C15	9.4 (6)	C13—C14—C15—C16	−177.7 (4)
O1—C13—C14—C19	−168.5 (4)	C13—C14—C19—C18	177.3 (4)
O2—C13—C14—C15	−172.8 (4)	C14—C15—C16—N7	179.7 (4)
O2—C13—C14—C19	9.4 (7)	C14—C15—C16—C17	−0.3 (7)
O8—C22—C23—C24	176.5 (5)	C15—C14—C19—C18	−0.6 (7)
O8—C22—C23—C25	−0.5 (7)	C15—C16—C17—C18	0.8 (7)
N1—N2—C3—C2	0.2 (5)	C16—N7—C21—O7	8.2 (7)
N1—C1—C2—C3	0.9 (6)	C16—N7—C21—C27^ii^	−171.9 (4)
N1—C1—C2—C4	179.0 (4)	C16—N7—C22—O8	−1.9 (6)
N2—N1—C1—C2	−0.8 (5)	C16—N7—C22—C23	176.2 (4)
N3—S1—N4—C8	0.2 (4)	C16—C17—C18—C19	−1.2 (7)
N4—S1—N3—C9	−0.5 (4)	C16—C17—C18—C20	178.2 (4)
N4—C8—C9—N3	−0.6 (6)	C17—C18—C19—C14	1.1 (7)
N4—C8—C9—C4	178.7 (4)	C17—C18—C20—O5	−174.2 (4)
N5—N6—C11—C10	−1.2 (6)	C17—C18—C20—O6	5.5 (7)
N6—N5—C12—C10	−0.3 (6)	C19—C14—C15—C16	0.2 (7)
N7—C16—C17—C18	−179.2 (4)	C19—C18—C20—O5	5.2 (7)
N7—C22—C23—C24	−1.6 (6)	C19—C18—C20—O6	−175.1 (4)
N7—C22—C23—C25	−178.6 (4)	C20—C18—C19—C14	−178.3 (4)
C1—N1—N2—C3	0.3 (5)	C21—N7—C16—C15	−90.6 (5)
C1—C2—C3—N2	−0.6 (5)	C21—N7—C16—C17	89.4 (5)
C1—C2—C4—C5	6.4 (8)	C21—N7—C22—O8	176.4 (4)
C1—C2—C4—C9	−173.6 (5)	C21—N7—C22—C23	−5.5 (6)
C2—C4—C5—C6	179.9 (5)	C22—N7—C16—C15	87.8 (5)
C2—C4—C9—N3	−1.0 (8)	C22—N7—C16—C17	−92.2 (5)
C2—C4—C9—C8	179.9 (4)	C22—N7—C21—O7	−170.1 (5)
C3—C2—C4—C5	−176.0 (5)	C22—N7—C21—C27^ii^	9.8 (6)
C3—C2—C4—C9	4.0 (8)	C22—C23—C24—C24^ii^	−175.5 (5)
C4—C2—C3—N2	−178.7 (5)	C22—C23—C24—C27^ii^	3.7 (6)
C4—C5—C6—C7	0.3 (9)	C22—C23—C25—C26	175.8 (4)
C5—C4—C9—N3	179.1 (5)	C23—C25—C26—C27	0.3 (7)
C5—C4—C9—C8	−0.1 (7)	C24—C23—C25—C26	−1.3 (7)
C5—C6—C7—C8	−0.3 (8)	C25—C23—C24—C24^ii^	1.5 (8)
C5—C6—C7—C10	180.0 (5)	C25—C23—C24—C27^ii^	−179.3 (4)
C6—C7—C8—N4	−178.3 (5)	C25—C26—C27—C21^ii^	−179.8 (5)
C6—C7—C8—C9	0.1 (7)	C25—C26—C27—C24^ii^	0.4 (7)
C6—C7—C10—C11	11.2 (8)	C28—N8—C30—O9	1.6 (9)
C6—C7—C10—C12	−168.5 (5)	C29—N8—C30—O9	−177.0 (5)

Symmetry codes: (i) *x*, *y*−1, *z*; (ii) −*x*, −*y*+2, −*z*+1; (iii) *x*, *y*+1, *z*.

### Poly[tetraaquabis[4,7-bis(1H-pyrazol-4-yl)benzo[c][1,2,5]thiadiazole][µ4-5,5'-(1,3,6,8-tetraoxo-1,3,6,8-tetrahydrobenzo[lmn][3,8]phenanthroline-2,7-diyl)diisophthalato]dinickel(II)] Hydrogen-bond geometry (Å, º)

**Table:**

*D*—H···*A*	*D*—H	H···*A*	*D*···*A*	*D*—H···*A*
O3—H3*A*···O2^iv^	0.87	1.92	2.758 (5)	162
O3—H3*B*···N6^v^	0.87	2.18	2.986 (5)	154
O4—H4*A*···O2	0.87	1.86	2.640 (4)	148
O4—H4*B*···O9^iv^	0.87	1.83	2.695 (5)	173
N2—H2···O9^iv^	0.88	1.92	2.779 (5)	166
N5—H5···N6^vi^	0.88	2.20	2.956 (6)	144
C1—H1···O10^v^	0.95	2.57	3.46 (3)	155
C11—H11···O1^v^	0.95	2.54	3.210 (6)	127
C11—H11···O6^vii^	0.95	2.49	3.279 (6)	141
C12—H12···O2^viii^	0.95	2.46	3.168 (6)	131
C28—H28*A*···N5^v^	0.98	2.65	3.426 (8)	136
C28—H28*B*···O4^iv^	0.98	2.72	3.414 (6)	128
C28—H28*C*···O8	0.98	2.26	3.157 (7)	151
O10—H10*A*···O7	0.87	2.08	2.95 (3)	180
O10—H10*B*···O6^vii^	0.87	2.20	3.01 (3)	155

Symmetry codes: (iv) −*x*+1, −*y*+1, −*z*+2; (v) −*x*+1, −*y*+1, −*z*+1; (vi) −*x*+2, −*y*+1, −*z*; (vii) −*x*+1, −*y*+2, −*z*+1; (viii) −*x*+2, −*y*+1, −*z*+1.
